# Effect of berberine on insulin resistance in women with polycystic ovary syndrome: study protocol for a randomized multicenter controlled trial

**DOI:** 10.1186/1745-6215-14-226

**Published:** 2013-07-18

**Authors:** Yan Li, Hongli Ma, Yuehui Zhang, Hongying Kuang, Ernest Hung Yu Ng, Lihui Hou, Xiaoke Wu

**Affiliations:** 1 Department of Obstetrics and Gynecology, First Affiliated Hospital of Heilongjiang University of Chinese Medicine, Harbin 150040, China; 2 Department of Obstetrics and Gynecology, University of Hong Kong, Hong Kong, China

**Keywords:** Berberine, Hyperinsulinemic-euglycemic clamp, Insulin resistance, Polycystic ovary syndrome

## Abstract

**Background:**

Insulin resistance and hyperinsulinemia play a key role in the pathogenesis of polycystic ovary syndrome (PCOS), which is characterized by hyperandrogenism, ovulatory dysfunction, and presence of polycystic ovaries on pelvic scanning. Insulin resistance is significantly associated with the long-term risks of metabolic syndrome and cardiovascular disease. Berberine has effects on insulin resistance but its use in women with PCOS has not been fully investigated. In this paper, we present a research design evaluating the effects of berberine on insulin resistance in women with PCOS.

**Methods/design:**

This is a multicenter, randomized, placebo-controlled and double-blind trial. A total of 120 patients will be enrolled in this study and will be randomized into two groups. Berberine or placebo will be taken orally for 12 weeks. The primary outcome is the whole body insulin action assessed with the hyperinsulinemic-euglycemic clamp.

**Discussion:**

We postulate that women with PCOS will have improved insulin resistance following berberine administration.

**Trial registration:**

This study is registered at ClinicalTrials.gov, NCT01138930.

## Background

Polycystic ovary syndrome (PCOS) is one of most common conditions affecting women of reproductive age and characterized by menstrual irregularities, signs of androgen excess, and, in many cases, obesity. Increased insulin resistance and compensatory hyperinsulinemia play a key role in the pathogenesis of PCOS [1,2]. Therefore, insulin-sensitizing agents have been studied in the management of PCOS.

Insulin-sensitizing agents include metformin and thiazolidinediones such as troglitazone, rosiglitazone, and pioglitazone. Metformin has been widely evaluated in women with PCOS, however, a recent meta-analysis showed that it is of no benefit in improving weight loss, insulin sensitivity, or lipid profiles in women with PCOS [3]. Moreover, it was also associated with a significantly higher incidence of gastrointestinal disturbance, leading to a higher dropout rate in clinical trials. Troglitazone, which underwent some trials for PCOS, was withdrawn from the market because of liver damage after both short- and long-term troglitazone treatment [4]. Although rosiglitazone and pioglitazone do not carry the same degree of risk of hepatotoxicity, these two drugs are classified as pregnancy category C drugs according to the Food and Drug Administration due to the potential risk of causing fetal growth restriction in animal experiments [5]. A high incidence of weight gain among the users further hampers their use in obese women with PCOS [6,7]. A Cochrane review indicated that insulin resistance in women with PCOS could be improved through lifestyle changes as well aspharmaceutical interventions [8].

Berberine, the major active component of *rhizomacoptidis*, exists in a number of medicinal plants and displays a broad array of pharmacological effects [9]. In Chinese medicine, berberine has long been used for its anti-diabetic effects. Recently, berberine has been shown to have positive effects on type 2 diabetes mellitus, insulin resistance, lipid metabolism, nitric oxide production, and metabolic syndrome [10-13].

The mechanisms of berberine in treating PCOS are still unclear. The beneficial metabolic effects of berberine in diabetic animals and type 2 diabetes mellitus patients are through the activation of AMP-activated protein kinase (AMPK) [11,14], which is similar to metformin. Metformin was reported to have a direct effect on steroidogenesis by ovarian cells in culture [15] and its ability to attenuate androgen production by ovarian theca cells is mediated through activation of AMPK [16]. In dexamethasone-induced insulin resistant theca cells and granulosa cells, this metabolic phenotype exaggerates androgenic potentials within these cells [17,18]. Berberine was found to improve insulin resistance in theca cells and granulosa cells in a way similar to metformin [19]. Therefore, we hypothesize that berberine could also have the same androgen production regulation effects as metformin within PCOS.

There is only one published study of berberine used in women with PCOS [20]. Eighty-nine Chinese PCOS women with insulin resistance were randomized into one of three treatment groups: berberine + cyproterone acetate (CPA) (n = 31), metformin + CPA (n = 30), and placebo + CPA (n = 28), for three months. Treatment with berberine + CPA in comparison to metformin + CPA showed a decreased waist circumference and waist-to-hip ratio (*P* <0.01), decreased total cholesterol (TG) and LDL-C (*P*<0.05), and increased HDL-C and sex hormone binding globuline (SHBG) (*P* <0.05). Similarly, treatment with berberine + CPA in comparison to placebo + CPA showed a decrease in wrist-to-hip ratio (WHR), fasting plasma glucose, fasting insulin, homeostasis model assessment for insulin resistance (HOMA-IR), triglyceride (TC), LDL-C, and TG (*P*<0.05) as well as an increase in HDL-C and SHBG (*P*<0.01). The author concluded that berberine, in comparison to metformin, showed similar metabolic effects presumably on amelioration of insulin sensitivity and reduction of hyperandrogenemia. Berberine also appeared to have a greater effect on the changes in body composition and dyslipidemia.

Berberine was used in combination with cyproterone acetate in the abovementioned study and its effect on insulin resistance in women with PCOS is still unclear. Therefore, the aim of this study is to compare the insulin resistance in PCOS patients with and without the use of berberine.

## Methods/design

### Study design

This is a multi-centered, randomized, double-blind, placebo-controlled clinical trial. Reporting of the study results will follow the CONSORT statement [21]. Subjects will be enrolled at four hospitals in mainland China: First Affiliated Hospital of Heilongjiang University of Chinese Medicine, First Affiliated Hospital of Harbin Medical University, Fourth Affiliated Hospital of Harbin Medical University, and First Affiliated Hospital of Guangzhou Medical School.

This study has been approved by the ethics committee of the First Affiliated Hospital of Heilongjiang University of Chinese Medicine (2010HZYLL-012). Informed consent will be obtained from each patient before any study procedure is performed according to good clinical practice. This trial has already been registered at clinicaltrial.gov, NCT01138930.

### Participants

A total of 120 subjects will be recruited at the clinics of the participating hospitals if they fulfill the inclusion criteria and do not have any exclusion criteria.

#### Inclusion criteria

1) Women aged between 18 and 35 years.

2) Confirmed diagnosis of PCOS according to the modified Rotterdam criteria and all subjects must have anovulation plus either polycystic ovaries and/or hyperandrogenism.

3) Body mass index equal to or greater than 23 kg/m^2^.

4) With no desire of children within 6 month.

PCOS is defined by the modified Rotterdam criteria: oligomenorrhea or amenorrhea, together with presence of ≥12 antral follicles (≤9 mm) and/or ovarian volume >10 mL on transvaginal scanning, and/or clinical/biochemical hyperandrogenism. Oligomenorrhea is defined as an intermenstrual interval >35 days and <8 menstrual bleedings in the past year. Amenorrhea is defined as an intermenstrual interval >90 days. Clinical hyperandrogenism in mainland China is defined with a Ferriman-Gallwey (FG) score ≥5 [22].

#### Exclusion criteria

1) Administration of other medications known to affect reproductive function or metabolism within the past three months, including oral contraceptives, GnRH agonists and antagonists, antiandrogens, gonadotropins, anti-obesity drugs, Chinese herbal medicines, anti-diabetic drugs such as metformin and thiazolidinediones, somatostatin, diazoxide, ACE inhibitors, and calcium channel blockers.

2) Patients with other endocrine disorders including 21-hydroxylase deficiency, hyperprolactinemia, uncorrected thyroid disease, suspected Cushing’s syndrome.

3) Patients with known severe organ dysfunction or mental illness.

### Interventions

Eligible patients will be randomized into one of the two arms: berberine (0.5 g, three times/day) or placebo. Berberine or placebo will be administrated orally for 12 weeks. Berberine and placebo were produced by the same pharmaceutical company (Renhetang Pharmaceutical Co., Ltd. China). They have the same appearance, size, odor and taste.

### Study specific visits and procedures

**Figure 1:**
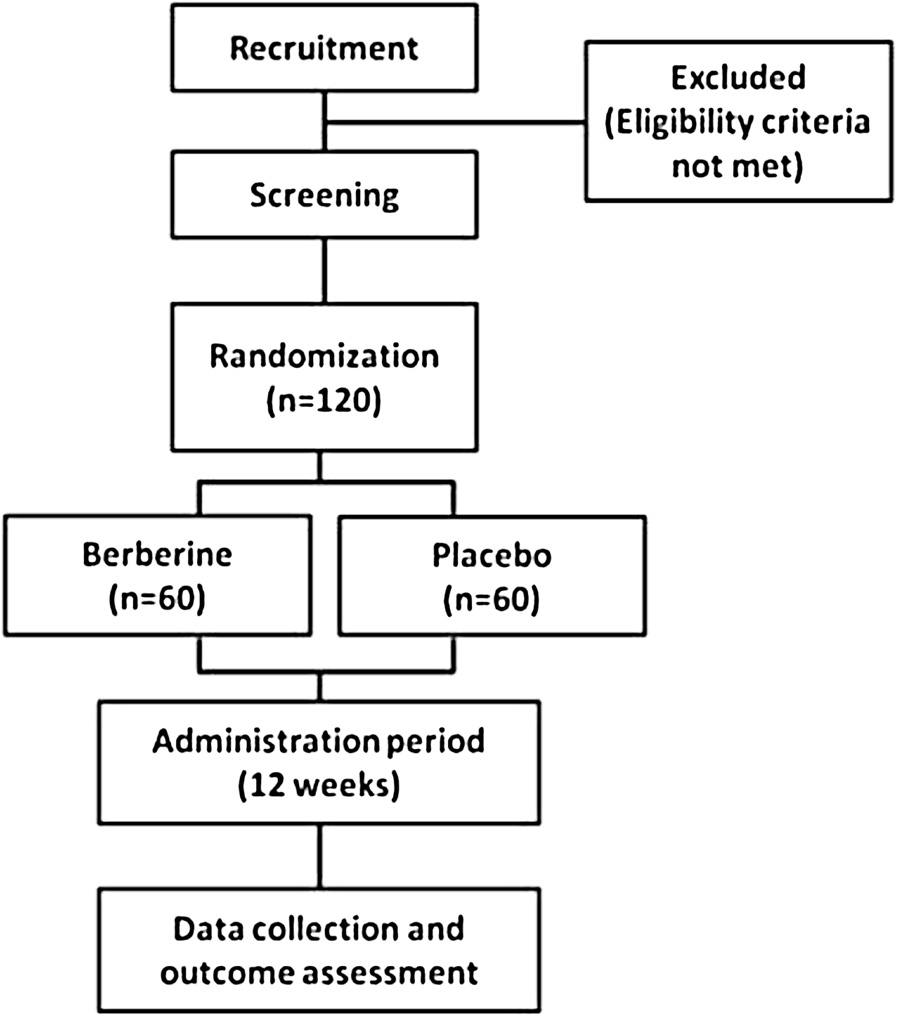
Study flowchart.

The trial has two phases: 12 weeks treatment with either berberine or placebo and three months follow-up (Figure 1).

Patients will attend six visits in total: screening visit, baseline visit, two monthly visits, end-of-treatment visit, and follow-up visit. Adverse events and concomitant medications will be recorded during visits. An overview of study visit is found in Table 1.

**Table 1:** Overview of study visits

	**Screening visit**	**Baseline visit**	**Monthly visit 1**	**Monthly visit 2**	**Monthly visit 3 and end-of-treatment visit**	**Follow-up visit**
Physical exam	X				X	X
Safety labs	X				X	
Transvaginal ultrasound		X			X	X
Fasting phlebotomy for study parameters		X			X	
Hyperinsulinemiceuglycemic clamp		X			X	
Query for adverse event and concomitant medications		X	X	X	X	X
Query for menstrual period	X	X	X	X	X	X
Medication dispensing and accounting		X	X	X		

Physical examination: Height, weight, hip and waist circumference, blood pressure, FG score, acne.

Fasting phlebotomy: Serum for the Central Core Laboratory.

Transvaginal ultrasound: endometrial thickness, ovarian volume, antral follicle count, and size of ovarian cysts or developing follicles.

### Randomization and allocation concealment

Subjects (n = 120) will be allocated randomly into one of the two groups in a ratio of 1:1. The identification code and random number, which are unique for each participant, were generated by an independent data center using SAS 9.2, seed = 20111024, block = 30, rand = 4. The investigators will get the randomization number through a web-based system operated by the independent data center (http://210.76.97.192:8080/xbj/). Subjects, investigators, and physicians treating the patients will be blind to the assignment.

### Outcome measurements

#### Primary outcome

Whole body insulin action: *in vivo* insulin action before and after treatment will be assessed with the hyperinsulinemic-euglycemic clamp. The glucose disposal rate (GDR; mg/(kg×min)), defined as the amount of glucose required to maintain stable blood glucose concentrations during the last 30 min of the clamping, will be used to define insulin action. The cut-off value of GDR in normal controls is (13.34 ± 1.41) mg/(kg×min) [23].

#### Secondary outcomes

1) Oral glucose tolerance test (OGTT): serum for glucose, insulin, and c-peptide levels will be determined.

2) Ovarian androgen biosynthesis: as measured by human chorionic gonadotropin (hCG), stimulated production of 17-hydroxyprogesterone (17-OHP), androstedione,and testosterone.

3) Hormonal profile including: testosterone, sex hormone-binding globulin (SHBG), follicle stimulating hormone (FSH), luteinizing hormone(LH), and dehydroepiandrosterone sulfate (DHEAS).

4) Fasting lipid metabolic profile: cholesterol, triglycerides (TG), high density lipoprotein cholesterol (HDL-C), and low density lipoprotein cholesterol (LDL-C).

5) Renal and liver function tests.

6) Weight, waist/hip circumference, blood pressure, FG score and acne before and after treatment.

7) Adverse events.

### Data entry and quality control of data

Case Report Forms (CRFs) will be developed for data entry and the electronic version will be implemented in a web-based data management system at http://medresman.org.

Quality control of data will be handled at three different levels. The first level is the real-time logical and range checking built into the web-based data entry system. The investigators at the participating sites are required to ensure the data accuracy as the first defense. The second is the remote data monitoring and validation that is the primary responsibility of the data manager and programmer at the Data coordination center (DCC). The data manager will conduct monthly comprehensive data checks, as well as regular manual checks (within the database system). Manual checks will identify more complicated and less common errors. The data manager will query sites until each irregularity is resolved. The third level of quality control will be the site visits, where data in our database will be compared against source documents. Identified errors will be resolved between the DCC and clinical sites. The visits will ensure data quality and patient protection.

### Sample size calculation and statistical analysis

We hypothesize that women with PCOS have an insulin resistance comparable to that of type 2 diabetes patients. According to previous results of berberine on type 2 diabetes [9], the GDR (the primary outcome) is 7.42 ± 2.37 mg/(kg×min) in the berberine group and 6.06 ± 2.21 mg/(kg×min) in the control group. The sample size was estimated according to the parameters: α = 0.05, β = 0.1, which results in 52/group. Considering a 15% drop-out rate over the course of the study, 120 total patients will be enrolled; 60 patients in each group.

Statistical analysis of all data will be performed by a dedicated statistician in a blind manner. One sample of the Kolmogorov-Smirnov test will be used to test the normal distribution of continuous variables. Continuous variables will be given as mean ± standard deviation if normally distributed, and as median (interquartile range) if not normally distributed. Statistical comparison will be carried out according to the intention to treat by student’s *t-*test, Mann–Whitney *U*-test, Wilcoxon signed ranks test for continuous variables, and χ^2^ test for categorical variables, where appropriate. All statistical analyses of the data will be performed using the SPSS program, version 16.0 (SPSS Inc., Chicago, IL, USA) and a *P* value <0.05 will be considered statistically significant. Subgroup analysis will be performed with respect to hyperandrogenism, which will be determined by evidence of hirsutism on physical examination or by hyperandrogenemia (biochemical elevations in total testosterone or free androgen index). Hirsutism in mainland China is taken as a FG score ≥5 [22]. Since cut-off value for testosterone varies between laboratories, hyperandrogenemia will be determined from local laboratories. Local cut-offs will be determined by each site prior to study initiation. The free androgen index (FAI) is calculated from measurable values for total testosterone and SHBG using the following equation: (FAI = Total testosterone in nmol/L/SHBG in nmol/L) × 100.

## Trial status

The study was conceived and designed in 2008. The first participant was randomized on December 25th 2011. At the time of manuscript submission, we have recruited 114 patients and the recruitment is ongoing.

## Abbreviations

AMPK: AMP-activated protein kinase; CPA: Cyproterone acetate; FG: Ferriman-Gallwey score; GDR: Glucose disposal rate; PCOS: Polycystic ovary syndrome; SHBG: Sex hormone-binding globulin.

## Competing interests

The authors declare that they have no competing interests.

## Authors’ contributions

XW, YL, and YZ conceived and designed the study. YL, XW, and EHYG drafted or critically revised the manuscript for important intellectual content. XW and EHYG read and approved the final manuscript. HM, HK, YZ, and XW participated in the acquisition of data, analysis and interpretation of data. XW had full access to all of the data in the study and take responsibility for the integrity of the data and the accuracy of the data analysis. All authors read and approved the final manuscript.

## References

[B1] Balen A Pathogenesis of polycystic ovary syndrome–the enigma unravels? Lancet 1999 354 966 967 10501353 10.1016/S0140-6736(99)00218-4

[B2] Diamanti-Kandarakis E Papavassiliou AG Molecular mechanisms of insulin resistance in polycystic ovary syndrome Trends Mol Med 2006 12 324 332 16769248 10.1016/j.molmed.2006.05.006

[B3] Tang T Lord JM Norman RJ Yasmin E Balen AH Insulin-sensitising drugs (metformin, rosiglitazone, pioglitazone, D-chiro-inositol) for women with polycystic ovary syndrome, oligoamenorrhoea and subfertility Cochrane Database Syst Rev 2012 5 CD003053 CD003053 22592687 10.1002/14651858.CD003053.pub5

[B4] Graham DJ Green L Senior JR Nourjah P Troglitazone-induced liver failure: a case study Am J Med 2003 114 299 306 12681458 10.1016/s0002-9343(02)01529-2

[B5] Yki-Jarvinen H Thiazolidinediones N Engl J Med 2004 351 1106 1118 15356308 10.1056/NEJMra041001

[B6] Baillargeon JP Jakubowicz DJ Iuorno MJ Jakubowicz S Nestler JE Effects of metformin and rosiglitazone, alone and in combination, in nonobese women with polycystic ovary syndrome and normal indices of insulin sensitivity Fertil Steril 2004 82 893 902 15482765 10.1016/j.fertnstert.2004.02.127

[B7] Ortega-Gonzalez C Luna S Hernandez L Crespo G Aguayo P Arteaga-Troncoso G Parra A Responses of serum androgen and insulin resistance to metformin and pioglitazone in obese, insulin-resistant women with polycystic ovary syndrome J Clin Endocrinol Metab 2005 90 1360 1365 15598674 10.1210/jc.2004-1965

[B8] Moran LJ Hutchison SK Norman RJ Teede HJ Lifestyle changes in women with polycystic ovary syndrome Cochrane Database Syst Rev 2011 6 CD007506 10.1002/14651858.CD007506.pub2 21328294

[B9] Birdsall TC Kelly GS Berberine: therapeutic potential of an alkaloid found in several medicinal plants Altern Med Rev 1997 2 94 103

[B10] Zhang Y Li X Zou D Liu W Yang J Zhu N Huo L Wang M Hong J Wu P Ren G Ning G Treatment of type 2 diabetes and dyslipidemia with the natural plant alkaloid berberine J Clin Endocrinol Metab 2008 93 2559 2565 18397984 10.1210/jc.2007-2404

[B11] Lee YS Kim WS Kim KH Yoon MJ Cho HJ Shen Y Ye JM Lee CH Oh WK Kim CT Hohnen-Behrens C Gosby A Kraegen EW James DE Kim JB Berberine, a natural plant product, activates AMP-activated protein kinase with beneficial metabolic effects in diabetic and insulin-resistant states Diabetes 2006 55 2256 2264 16873688 10.2337/db06-0006

[B12] Xu MG Wang JM Chen L Wang Y Yang Z Tao J Berberine-induced upregulation of circulating endothelial progenitor cells is related to nitric oxide production in healthy subjects Cardiology 2009 112 279 286 18815446 10.1159/000157336

[B13] Affuso F Mercurio V Ruvolo A Pirozzi C Micillo F Carlomagno G Grieco F Fazio S A nutraceutical combination improves insulin sensitivity in patients with metabolic syndrome World J Cardiol 2012 4 77 83 22451856 10.4330/wjc.v4.i3.77 PMC3312235

[B14] Yin J Xing H Ye J Efficacy of berberine in patients with type 2 diabetes mellitus Metabolism 2008 5 712 717 18442638 10.1016/j.metabol.2008.01.013 PMC2410097

[B15] Mansfield R Galea R Brincat M Hole D Mason H Metformin has direct effects on human ovarian steroidogenesis Fertil Steril 2003 97 956 962 12749437 10.1016/s0015-0282(02)04925-7

[B16] Will MA Palaniappan M Peegel H Kayampilly P Menon KM Metformin: direct inhibition of rat ovarian theca-interstitial cell proliferation Fertil Steril 2012 98 207 214 22608319 10.1016/j.fertnstert.2012.04.010 PMC3389190

[B17] Wu X Theca insulin resistance: dexamathasone induction and berberine intervention Fertil Steril 2010 94 s197

[B18] Zhao L Li W Han F Hou L Baillargeon JP Kuang H Wang Y Wu X Berberine reduces insulin resistance induced by dexamethasone in theca cells in vitro Fertil Steril 2011 95 461 463 20840879 10.1016/j.fertnstert.2010.07.1090

[B19] Wu X Yao J Hou L Kuang H Berberine improves insulin resistance in granulosa cells in a similar way to metformin Fertil Steril 2006 86 459 460

[B20] Wei W Zhao H Wang A Sui M Liang K Deng H Ma Y Zhang Y Zhang H Guan Y A clinical study on the short-term effect of berberine in comparison to metformin on the metabolic characteristics of women with polycystic ovary syndrome Eur J Endocrinol 2012 166 99 105 22019891 10.1530/EJE-11-0616

[B21] Schulz KF Altman DG Moher D CONSORT group CONSORT 2010 statement: updated guidelines for reporting parallel group randomized trials Ann Intern Med 2010 152 726 732 20335313 10.7326/0003-4819-152-11-201006010-00232

[B22] Zhao X Ni R Li L Mo Y Huang J Huang M Azziz R Yang D Defining hirsutism in Chinese women: a cross-sectional study Fertil Steril 2011 96 792 796 21762890 10.1016/j.fertnstert.2011.06.040

[B23] Cheng Q Li Q Bai X Feng J Huang J Liu X Establishment of botnia clamp technique in healthy women J of Chongqing Med University 2005 30 598 600

